# Supplementary material for: Gross Chromosomal Rearrangements in Kluyveromyces marxianus Revealed by Illumina and Oxford Nanopore Sequencing
Source: Int J Mol Sci. 2020 Sep 26;21(19):7112. doi: 10.3390/ijms21197112 (PMC7582981; doi:10.3390/ijms21197112)
Supplement: Supplementary file 1 [file ijms-21-07112-s001.pdf]

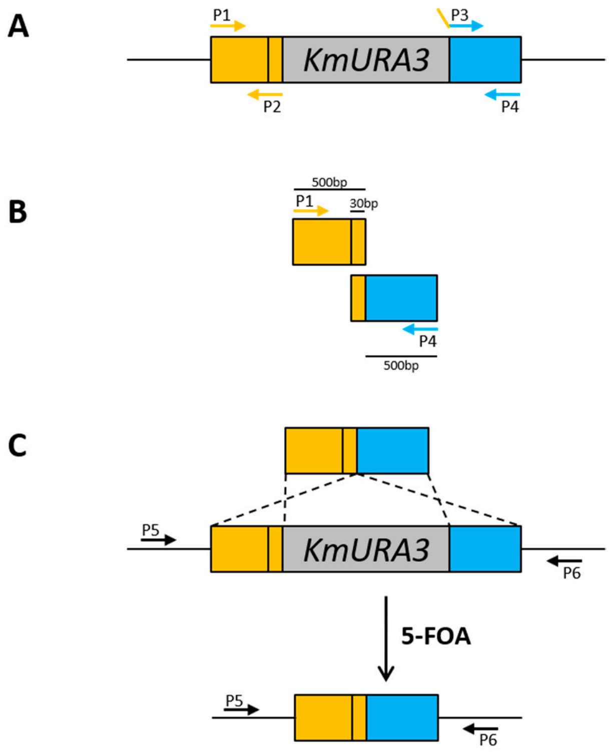

Figure S1: Construction of G13. (A) Genomic DNA was used as a template to amplify 500bp targeting arms flanking the URA3 ORF of *K. marxianus*. P1 and P2 were used to amplify the left arm, while P3 and P4 were used to amplify the right arm. (B) In the second round of PCR, the left and right arm was joined by 30bp overlap using P1 and P4. (C) The final PCR of a total volume of 600µl (1x Phusion master mix, 1µl of prior PCR products, and 0.2µM of each primer) was purified yielding 18µgs after gel purification (Qiagen Qiaquick). Either 1 or 5µgs of the targeting construct was electroporated into K. marxianus. The cells were grown to mid log phase, washed several times with ice cold water and further treated with LiAC and DTT at 30C for 45 min (final concentrations of 80mM LiAC, 20mM DTT, 10mM Tris-HCl (pH 8.0), 1mM EDTA). Cells were further washed with ice cold water and resuspended in 1M sorbitol in water as an electroporation buffer. One hundred µls of cells were mixed with DNA in a 0.4mm gap electroporation cuvette and pulsed at 2.5kV, 25µF, and 200 ohms in a BioRad Gene Pulser Xcell electroporation apparatus. Cells were recovered in YPDS (YPD plus 1M sorbitol) overnight at 30°C and spread on 5-FOA plates the next day (SDA-Ura Powder, 500mls, Sunrise Science products catalog number 1704-300, plus 25mg uracil, Sunrise Science products catalog number 1906-10, and 0.5 g of 5-FOA, Gold Biotechnology catalog number F-230-10). P5 and P6 were primers used for screening positive transformants based on size differences.

TGAGAGTGCACCACGCTTTTCAATTCAATTCATCATTTTTTTTTTATTCTTTTTTTTGATTTCGGTTTCTT
TGAAATTTTTTTGATTCGGTAATCTCCGAACAGAAGGAAGAACGAAGGAAGGAGCACAGACTTAGA
TTGGTATATATACGCATATGTAGTGTTGAAGAAACATGAAATTGCCCAGTATTCTTAACCCAACTGCA
*CAGAACAAAAACCTGCAGGAAACGAAGATAAATC*__**ATG**__**TCGAAAGCTACATATAAGGAACGTGCTG**
CTACTCATCCTAGTCCTGTTGCTGCCAAGCTATTTAATATCATGCACGAAAAGCAAACAAACTTGTG
TGCTTCATTGGATGTTCGTACCACCAAGGAATTACTGGAGTTAGTTGAAGCATTAGGTCCCAAAAT
TTGTTTACTAAAAACACATGTGGATATCTTGACTGATTTTTCCATGGAGGGCACAGTTAAGCCGCT
AAAGGCATTATCCGCCAAGTACAATTTTTTACTCTTCGAAGACAGAAAATTTGCTGACATTGGTAA
TACAGTCAAATTGCAGTACTCTGCGGGTGTATACAGAATAGCAGAATGGGCAGACATTACGAATG
CACACGGTGTGGTGGGCCCAGGTATTGTTAGCGGTTTGAAGCAGGCGGCAGAAGAAGTAACAAA
GGAACCTAGAGGCCTTTTGATGTTAGCAGAATTGTCATGCAAGGGCTCCCTATCTACTGGAGAATA
TACTAAGGGTACTGTTGACATTGCGAAGAGCGACAAAGATTTTGTTATCGGCTTTATTGCTCAAAG
AGACATGGGTGGAAGAGATGAAGGTTACGATTGGTTGATTATGACACCCGGTGTGGGTTTAGAT
GACAAGGGAGACGCATTGGGTCAACAGTATAGAACCGTGGATGATGTGGTCTCTACAGGATCTGA
CATTATTATTGTTGGAAGAGGACTATTTGCAAAGGGAAGGGATGCTAAGGTAGAGGGTGAACGT
TACAGAAAAGCAGGCTGGGAAGCATATTTGAGAAGATGCGGCCAGCAAAAC__TAA__aaaactgtattataa
gtaaatgcatgtatactaaactcacaaattagagcttcaatttaattatatcagttattaccctg

Figure S2: Sequence of *ScURA3* used in transformation

Bold: *ScURA3* ORF. Underscore: Start and Stop codons. Primers used for *ScURA3* amplification are in blue.

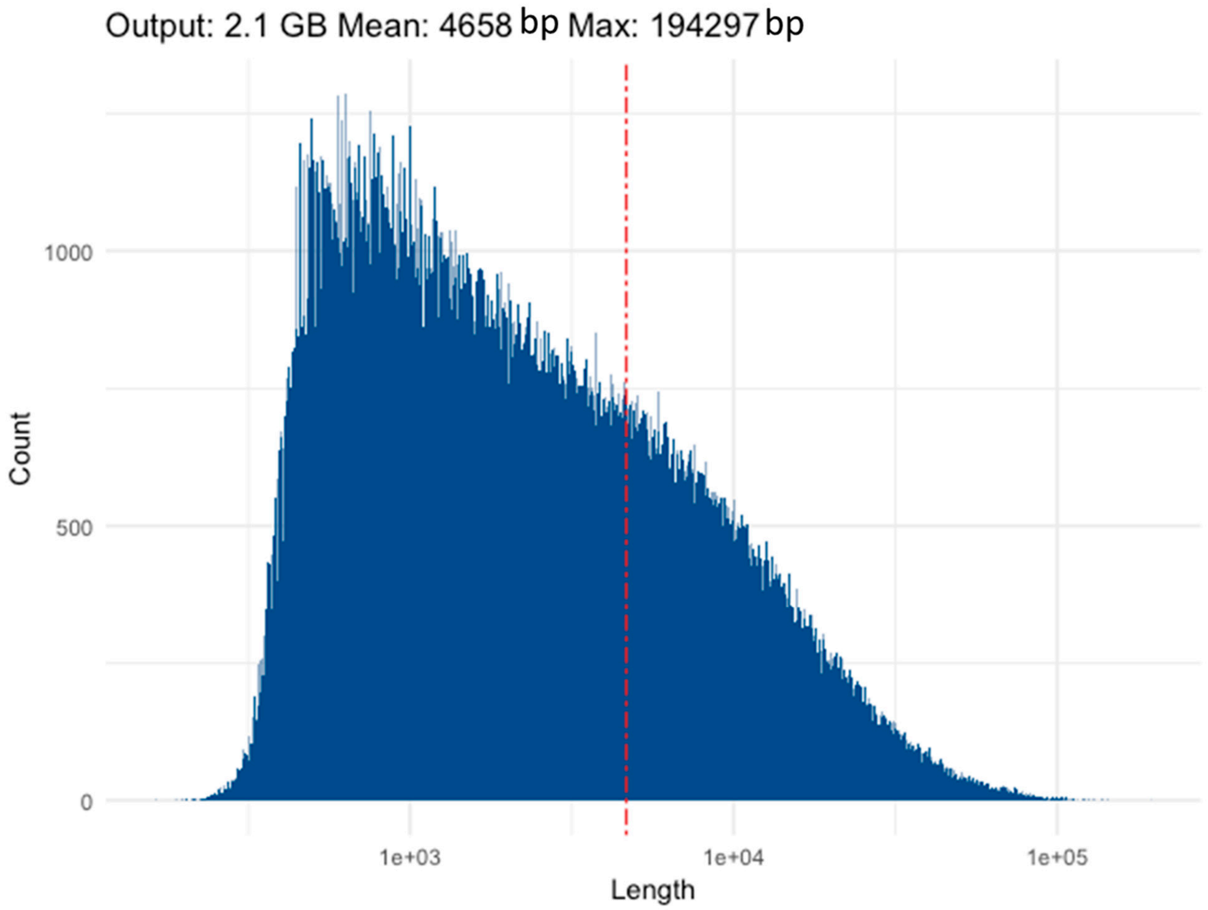

Figure S3. Distribution of Oxford Nanopore sequencing read lengths for the 24-clone pool DNA of *K. marxianus ScURA3* cassette DNA transformants.

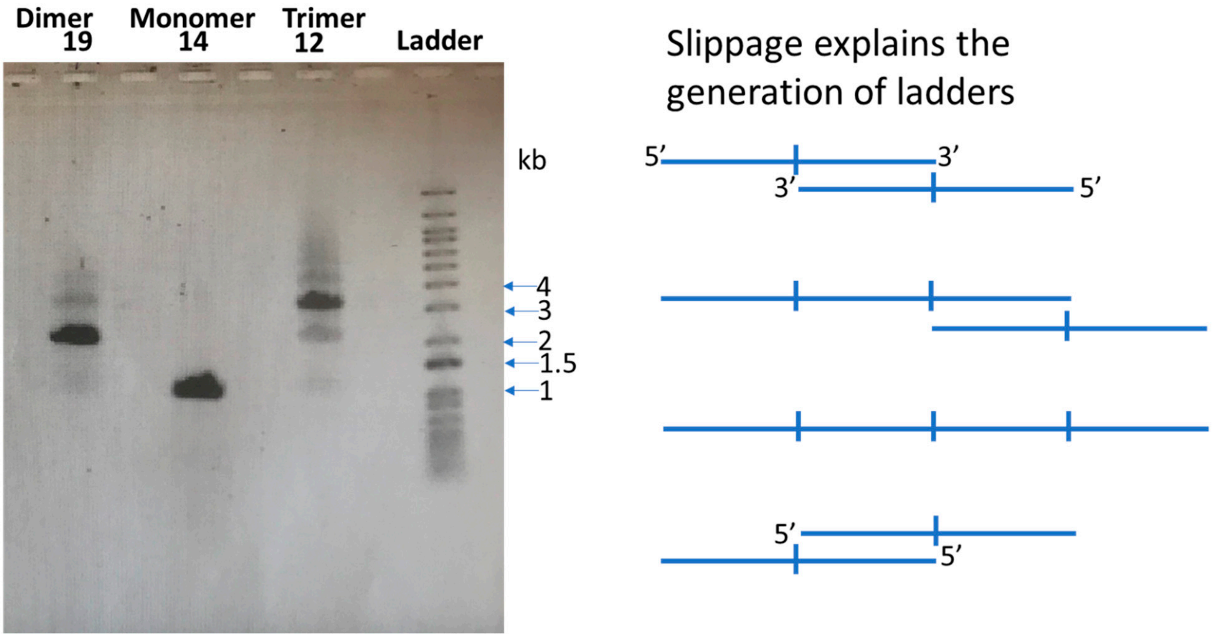

Figure S4. Gel electrophoresis of PCR amplified *K. marxianus* ura3 monomer (clone 14), dimer (clone 19) and trimer (clone 12) transformants using flanking primers. The dimer and trimer lanes show ladders of bands produced by slippage during the annealing step of each cycle.

Clone 6 PS9 intergenic-intergenic
PS15 for deleted region clone6

AAAACCAAAAAAAACCATAAAAACCAAAACA TGAGAGTGCACCACGCTTTTCAA

chr4 714164mi

CAATTTAATTATATCAGTTATTACCCTG AACGACTTGATGATGTACATAG

chr4 762150mi

Nanopore monomer out2 read 005,117,134(both ends), 320(both ends), 538(both ends), 597(both ends), 688(both ends)

> read134

AAAACATCCGCACCTGCAAATTACGCCTTCCAGTGACTGTCTAATCGCTCAACAAACTCCTCTCC
CACTGTCTCACGATACTCGACTTTGTTGCGATGAGCATAGGTTTCGGGAATTTCAATGGATGTATA
TCTGTGTACTTAACGCGATATTTAAATCACGTGTAAGGAAAAAACCAAAAAATATAAAAAGCAA
AACA chr4 714164

TGAGAGTGCACCACGCTTTTCAATTCATCATTTTTTTATTCTTTTTTGATTTCAGTTTTCATGAAA
TTTTTTGATTCGGTAATCTCCGAACAGAAGGAAGAACGAAGGAGCACAGACTTAGATTGGTATAT
ATACGCATATGTAGTGTTGAAGAAACTAAAATTGCCCAGTATTCTTAATAACTGCACAGAACAA
AAACCTGCAGGAAACGAAGATAAAATCATGTCAGAAAGCTACATATAAGGAACGTGCTGCTACT
CATCCTAGTCCTGTTGCTGCCAAAGCTATTTAATATCATGCCACGAAAAGCAAACAAACTTGGCC
ATTGGATGTTCGTACCACAAGGAATTACAGATTGGTTGGAAACGTGGGTCCCAAAATTTGTTTAC
TAAAACACATGTGGATATCTTGACTGATTTTCCATGGAGGGCACAGTTAAACCGCTAAAGGGCAT
TTATCCGCCAAGAAATTACAGTGCACTCTTCGAAGACAGAAAGTTGCTGACATTGGTAATACAGT
CAAATTGCAGTACTCTGCGGGTGTATACAGAATAGCAGAATGGGCAGACGTGGAATACACGTTG
TGGTGAGCCAGGTATTAGCGGTTTGAAGCAGGCGGCAGAAGAAGTAACAAAGGAACCTAGAGG
CCTTTGATGTTAGCAGAATTGTCATGCAAGGGCTCCTCTGCTGGAGAATATACTAAGGGTACTGTT
GACGTGCGAAAGAGCGACAGATTTTGTTATCGGCCTTTATTGCTCAAAGAGACATGGGTGGAAA
GATGAAGGTTACGATTGGTTGATTATGACTTGGTGGCGTTTAGGTGAACAGGGGGAGCGCATTGG
GTCCAACAGTATAGAACCGTGGATGATGTGGTCTCTACAGGATCTGACATTATTATTGTTGGAAA
GAGGACTATTTGCAAAGGGAAGGGATGCTAAGGTAGAGGGTGAACGTTACAGAAAAGCAGGCT
GGGAAGCATATTTGAAGAAGATGCGGCCAGCAAAATAAAAGGCGGTATAGTAAGAAATGCATG
TATACTAAACTCACAAATTAGAGCTTCAATTTAATTATATCAGTTATTGCCTG
chr4 762150
AACGACTTGATGATGTACATAGACTTCTGTAGGAGATTTACTGGATACACAGGATGGGGTGGTAA
AAGGGCTGGGAGATAATTTATTGGCTTTTACAGAGCCCGGTAGATCAAGATTCCGTAGTGTGTGA
GGGTAAATAAGTAAACAACCGGACGGGTTGGCGTATAGTAGAGAGTAAGTAGTTGTGATAGCAC
T

Yellow highlight indicates the flanked the *ScURA3* fragment

Figure S5. Clone 6 Oxford Nanopore sequencing read

MiSeq (mi), Nanopore (nox)

Clone 7 **PS7** **intergenic-intergenic**

AATCAGAATCTTGAAGAGGAAAAGCATACCA TGAGAGTGCACCACGCTTTTCAA chr3 953765 mi

CAATTTAATTATATCAGTTATTACCCTG ATGCTGGCATGAGGGGGGGAAGTC chr3 824416 mi

Nanopore monomer out2 read 594, 131(both ends),407(both ends),442(both ends),506(both ends),594(both ends),715

> read131

TCATCGGAAGAAGATCGGAACGGCTGCAGGTTGTATCGATTTTGTATTGGCTTGCATTGTTATGCG
TGTTTTGAGTTTTGCGCTGGAATGGTGCATTACCCTTCGTGGGCTTCTATTATTATTTGTTTCCTTAT
AATAGCGATATTTGTTGCATGTTTCTTGTACATCAGATCAAGTCTTGAAGAGGAAAAGCATACCA

chr3 953765

TGAGGTGCACCACGCTTTTCAATTCATCATTTTTTTTTTTTATTCTTTTTTGATTTCGGTTTCCTTGAA
ATTTTTGATTCGGTAATCTCCGAACAGAAGAACGAGAAGAACACAGACTTAGATTGGTATATATA
CGCATATGTAATTTGAAGAAACATTAGAAATTGCTTGATATTCTTAACCCAACTGCACACAAGAC
AAAGAGCAGAACGAGATAAATCATGTCAGAAAGCTACATATAAGGAACGTGCTGTACTCATCCC
TAGTCCTATTTGCTGCCAAACTATTTAATATCATGCACGAAAAGCAAACAAACATTTCATTGGAT
GTTCGTACCACCAAGGAATTACTGGAGTTCGGTTGACATTGGGTCCCAAAGTTTGTTTTACCTAAA
ACACATGTGGATATCTTATTCAGTTTTTCCATGGAGGCACAGTTAAGCCGCTAAAAGGCATTACC
TCCGCCAAGTACAATTTTTTACTCTTCGAAGACAGAAGGATTTGCTTTGACATTGGTATTACAGTC
CAGAATTGCGGTACTCTGCAGATTGGTATACAGAATAGCAGAATGGGCAGGGCATTACAAGAAT
ACAGTGGTGAGCCCAGAGTATTATTAAGCGGTTTGAAGCAGGCGGCAGAAGAAGTAACAAAAG
GAACAGAGGCCTTTTGATGTTATAGAATTAATTGTCATACAAGGAGCTCCCTATCTACTGGAGAA
TATACTAGGGTACTGTTGACATTGCGAAAGAGCGACAAAGGAATTTTGTTATCAGCTTTATTGCTC
CAAAGAGACATAGATTGGAAAGATAGAAGGTTACGATTGGTTGATTATGACACCCGGTGTGAGT
TTAGATGACAAAGGGGAGCCGCATTAGGTCAACAGTATAGAACCGTGGATGATGTGGTCTCTGG
GATCTGACATTATTATTGTTGGAAGAGGACTATTTGCAAAGGGAAGGGATGCTAAGGTAGAGGG
GTGAACGATTACAGAAAAGCAAGAGCTGGGAAGCATATTTGAAAGATGCGTAGCAAAACTAAA
AACTGTATTATAGAAATGAAATGCATGTATACTAAACTCACAAATTGAAACTTCCAAGATTTAAT
TATATCAGTTATTACCTG

chr3 824416

ATGCTGGCATGAAGTCAGTGCTATATATATGTTTGTACTACGCTATGATGGGTACGTGGAGGTGT
GTGTGAAAGGCGCGCTGGCCAGACCCATCGCCCTGATTTCCAACTAGCCCAGCCCCTAGAGCCC
CATCCATCATTGTTGGATCAGCACCAACACCCATCCACCATGATCTTGTATACGTATACGTTTTGA
AGC

Yellow highlight indicates the flanked the *ScURA3* fragment

Figure S6. Clone 7 Oxford Nanopore sequencing read

MiSeq (mi), Nanopore (nox)

Clone 8 PS5 **SG4EUKG584656 -intergenic**

TCCAAAGTCAAACCTTCCAAGTCGACG TGAGAGTGCACCACGCTTTTCAA chr1 377332 mi

CAATTTAATTATATCAGTTATTACCCTG GTAATGCAAATTTATACAGGTCAAA chr1 375438 mi

Nanopore monomer read283 (read 283 is a ura3 monomer followed by non ura3 and then followed by 900 bp ura3 missing 200 bp of head end)

PS5a TCCAAAGTCAAACCTTCCAAGTC(GACG) chr1 377332 mi
PS5b TTTTGACCTGTATAAATTTGCATTAC chr1 375438 mi
1894 bp inversion size
With inversion, the PCR product is ura3 size of about 1.2kb.
CAATTTAATTATATCAGTTATTACCCTG GGGCAGCAGTAGTGGATATGCAGC chr1 375422 mi,nox
Read 283(both ends)
Nanopore monomer read468(tail end),649 (??one end 377318),708b, 175
> read283 chr1 377460 PS 283top
ATAGACTCAATCCTGACGGTACCGTTGACTGTGTTTTGGGAAGGGTTTGGATCATGGATCTGACG
TATTCACTCCTGGAGTGAAGTAGGAACCGTTGTGCAAGAGTCTGGAGTAAGTTGGGGTGACAATT
GGTCTCTCAAGTGGCGAGACTTCCTTGTCCAAAGTCAAGCCTTCAAGTCTGACG PS5a>

TGAGTGCACCACGCTTTCAATTCATCATTTTTTTATTCTTTTTTGATTTCGGTTTTCCTTGAAATTTT
TGATTCGGTAATCTCCGAACAGAAGAGACGAAGGGAAGGCACGGAGCTTAAATTGGGTATATAT
ACGCATATGTAGTGTTGAAGAAGCGTGAAATTGCCCAGTATATTCTTAATAGCACGCAGGGCAA
AAACCTTGCAGGAAACGAAGATAAATCATGTCAAGCTACATATAAGGAACGTGCTGCTACTCAT
CTAGTCCTGTTGCTGCCAAGCGTTTAATAATGCACGAAAAAGCAAAACAAACTTGTGTACTTCAT
TGATGTTCCGTACCACCAAGGAATTACTGGAGTTAGTTGAAGCATTAGGTCCCAAAATTTGTTTA
CTAAAACACGTGGATATCTTGACTGATTTTTCCATGGAGGGCTGGATTCGGCACTTCAAAGGCA
TTATCGCCGTACAATTTTACTCTTCGAAGACAAGAAAATTTGCTGACATTAGTATACAGTCAAATT
GCAGTACTCTGCGGGTGTATACAGAATAACCAAGAATGGCAGACATTACGAATGCACACGGTG
TGGTGGGTAGGTATTGATGTTAGCGGTTTTGAAGCAGGCGGCAGAAAGAAGTAACAAAAGACAC
AGAGGCCTTTTGATGTTGGGAATTATGTATTACAAAGAGGCTCCCTATCTGGAGAATATACTAAG
GAGTGCTGTTGACATTGCGAAGAGCGACAAAAGATTTTGTTATCGGCTTTATTGCTCAAAGAACA
TGGTGAAGATAGAAAGGTTACGATTGGTTGATTATGACACCCGGTGTGAGTTTTAGATGACAAAG
GGGAACGCATTGGGTCAACGAGTATAGAACCGTGGATGATGTGGTCTCTGGGATCTGACGTGGC
AGTATTTGAAGAGGACTATTTGCAAAGGAGGTGCTAGGGTAGAGAGATTGAACGTTACAGAAGA
AAAGCAAGCTGGGAAGCATATTTGAAGATGCGGTATGTAAAGAGCTAAAAAACTGTATTATAAG
TAAATGCATGTATGCTAAACTCACAAATTAGAGCTGTTTAATTATATAGTTATTACCCTG
Chr1 375438 PS5b<
GTAATGCAAATTTATACAGGTCAAAAGGCGATCCTTAACAGGTCGCGCGCTCGCTCTAACTCAAT
TTTTTTTTTTATTCCGCACCGCACACAAGATTTACTCTCTGTGGCCATTAATGCCTAAACTGGAAA
CTCTCCCTTTCTAAGGCATAAGGCATTCAAAAGAAACGATATTGCAACCCGTACACCTGGTTTTA
TTTCTTTTTCTTGTTTTTCCTGCTTTCCCTATTTACAACCGGGTAACCTCGTCTTCTCTTGCACTTAGA
GAACACAAGCCAAAAACGCTGAAGCGCAAGAACACAAGAAACCCCAGCATCAGCCAGTTATTG
TCAAGCTAATTGAAAGTAGCGATCGTCCTTGTGACTACCGCCATAATATTCGTGATGATTCAAAA
CCTCAAAGATGAGCCAGTTTGGAACCTTGGAAACTTTAATTCAGGGTTTTCCAGCACAGTTTAATT
TACAGTTTTAGTGATATATTTTGGACCGCGATGGAAATCTTTTTTTTCAATTCACTTGTTTTTAAATT
TCTGTGAGTGTATTTTAGTTATGGATGGGTCACCACCAAAACAACAATTTCACAGAGGTGGGCAT

TTGGGCTCTAAGTGCTAGTCGTCAGTGTAGGGATATGAGTCATTTTTATTTTTCCTTTCGCTGTCTT
GCTTGTTTGAGTGTAGGTAAGAGTCAGAAACCAGTGATACAGACTAATGCTGTAGCATTGCCTGT
TGATAAAATTAATTAAGATGCCCATCGAAAATGAGTAAAAGTTGGAGAAAACTTTAGACGGTAT
ATAAATGGTTCGGAGAGTGGAACAAATATGTGGAGGAAGTAAAGTTCTCTTGCGTATGGTTGTAA
TAAGCTAATGTATATATTTGTTAGTGTGTATAGAAGCTAAGCAATGTAGATTGATAGTGGAAGGA
TAGTTGCAACAGGGAAGAGCGATTTCACGCAAGAGCATAGATCTAAGTAGTTTACAAAAATGTC
TAAAAGGTAAGGTTTGTTGGCATACTCCGGTGGTTGGAATACCTCTGTGATTTTGGCGTTGTTGGA
ACAGGAATTGCAAAGTTGTTACCATTTGCAGCCAATGTTGGTCCAGAAGACTGAGGCCGCGAAA
ACTTTGCTTGTTGGTGCCACCAAAGTTTGTTCTAGTGGACTGAGAGAAGAAATTTTCAGGACGTTT
TGTTCCAGCTTCTGTGCAAGTTAACGCTTTGTTATACGAATGTGTACCTGTGGAACATCTTTGTGA
ACCAGTGATTGCCAAGGCCCAAATTGACGTGGCCAAGCAAGAGTTGTTTGCCGTTGCGCACGGG
TGTACCGGTAGGGTAACGATAAAATCAACGTTTGGAACTAGGGTTCTACGCCTTGAAGCCAGAC
GTCCCAGTCATTGCTCCATGGAGAGACCCATCTTTCTTCAACTTAGATTTTGCTGGTAGAAAGGAT
CTTTTTGGCGCTGCTGAAAGACATTCCAGTGGTGCAAACTGCTTCAAGCCATGGTCTACCGATGA
GAACTTGGCCCACATACCACGAAGCCGGTATTCTAGAAGATCGACCAAAACCACCCAAAGGAC
ATGTGGAAGTTGATCACCGACCCACAAGATGCCCCAGACCAACCACAAGACTTGGACATTGGGC
GAAAAGGGTTTGCCAGTCAGAGGTTGGCGTACAAGGATGCCAGGTCGGTAAAGGGAAATCACA
GCCACAACCCATTGGACATTTCTTGACCGCTGCCAAGTTGGCCAGAGCTAACGGTGTTGGCAGAA
TCGAGCATTGTCGAAAACCGTTACATT

AACCTAAAGTCCAGAGGCTGTTACGAACAGAAATGTTGTCGTCTCTTGGCAACTGCACAGAACA
AAACCTGCGGGAAACCGAAGATAAATCATGTCGAAGCTGCATATAAGGAACGTGCTGCTGCGCA
AATGCGATCCTGTTGCTGCCAAGCTGTTTAATATCATGCACGAAAAGCGGCAAAACTTGTGTGCT
TCGTGGATGTTCGCTCCACCAAGGAATTACTGGAGTTAGTTGAAGCATTAGGTCCCAAAATTTGT
TTACTAAAGCACATGTGGATATCTTGACTGATTTTTTTCCTGTGGAGAACTGGATTAAAGCCGCTA
AAAGGCATTATCCGCCAAGTACAATTTTACTCTTCGAAGACAGAAAATTTTGCTGACATTGGTAA
TACAGTCAAATTGCAGTACTCTGCGGGTATACAGAATAGCAGATGGGCAGACATTACGAATGCA
CACACGGTGTGAGGTGGGCCGAGGGCAGTATTGCTTGGTTTTGAAGCAGGCGGCAGAAGAAGTA
ACAAAGGAACCTGAGGCAGCACAGTGTTAGCAGAATTGTCATGCAAGGGCTCCCTATCTACTGG
AGAATATACTAAAGAATTTACTGTTGACATTGCGAAACGACAAAGATTTTGTTATCATTTATTGCT
CAAAGAGACATGGGTGGAAAGAGATGAAGGTGCATTGGTTGCGGCCACCCGGTGTGGGTTTAGA
TGACAAAGGGAGACGCATTTGGGTCAACAGTATAGAACCGTGGATGATGTGGTCTCTACAGGAT
CGACATTATTATTGTTGGAAGAGGACTGTTGCAAAGGGATGCTAAGGTAGAGAGATTGAACGTT
ACAGAAAAGCAGGCTGGGAAGCATGTTGAGAGAATGCGTAACAAACTAAAAACTATGTTATAA
GTAAATGTATGTACTAAACTCACAAATTAGAGCTTCAATTTAATTATATCAGTTATTACCCTG
Chr1 375422 PS6b<
GGGCAGCAGTAGTGGATATACAGCTGTCCCGGGCGTTAGAGTAAAAAATTAGAGTGGAAGAATA
GAAAAAGAAAGAATATTGTGTAACGTTTGCAGTATCTATTTATCTTATGTAGATGGGTAAAGCTT
TTAGTTTATTTGTCCGATGACAGCTAGCTCTCGCTAGTCGTATGTGATTTTCTCGAATATCTTGCTT
CAAT PS 283 bottom

Yellow highlight indicates the flanked the *ScURA3* fragment
Green highlight indicates partial ura3_TestF

Figure S7. Clone 8 Oxford Nanopore sequencing read
MiSeq (mi), Nanopore (nox)

Clone 5 PScl5 intergenic chr7--intergenic chr6 Dimer

Miseq

TAACTTCGGAATATTGAATTAATCGTCTCC TGAGAGTGCACCACGCTTTTC chr7 24526 mi

CAATTTAATTATATCAGTTATTACCCTG TATATTATATTAAATAAAAATAAT chr6 375003 mi

Found in chr6 Nanopore assembly, but not in MiSeq assembly

Nanopore sequence read 17015, 008a,

302,311,329,355,383,390,439,480,532,550,563,581,622,626,629,668,678,685,712,721,724

>read355 reverse complement

TGCTGGACTAACTATAGGGTAGTTCGTAGCTGTGATCTTGATTGTCAGTTTTGCTCAAATAAATAT
TATATATATATATAGAAAGAAGTGGGTAGACCACATTGTTTACAATACGTCTTAAATTGTGTTCG
ATGAGCATGTAGACAAAATTAGAGTAGAAGTGTTGGGTTCTAACTTCGAATATTGGAATTAATCG
TTCC **chr7**

GAGAGTGCGCACCACGCTTTTCAATTCATCATATTTTTTATTCTTTTTGATTTCGGTTTCTTGAAA
TTTTTTTGATTCGGTAATCTCCGAACAGAAGGAAGAACGAAGGAAGGAGCACAGACTTAGATTG
GTATATATACGCATATGTAGTGTTGAAGAAACATGAAATTGCCCAGTATTCTTAACCCAACTGCA
CAGAACAAACCCGCAGGAAAACTGAAGATGCTAAATCATGTCGAAAGCTACATAAGGAACGTG
CTGCTACCATCCCAGTCCTGTTAAGCTGCTGCCATTTATCATCATGCACGAAAAGCAAACAACTT
GTGTGCTTCATTGGATGTTCGTACCACCGCTGAACTACTGAGTTAGTTGAAGGCCATTAGGTCCCA
AAATTTGTTTACTAAAACACACATGTGGATATCTGACTGATTTCTTTCCATGAGGGCACAGTTAAG
CCGCTAAAGGCATTATCCGCCAGCACAATTTTTACTCGAAGACAAGAAATTTGCTGACATTGGTA
ATACAGTCAAATTGGTAGTACTCTGCGTGGTAGTATACAGAATAGTGCAGAATAAGACATTACG
AATGCACACGGTGTGGTGGGCCCAGGTATTGTTATGGTTTGAAGCAGGCGCGGTAGAAGTAAAT
GAAACCTAGAGGCCTTTGATGTTAGCAGAATTGTCATGCAAGGGCTCTCATTCTACTGGAGAAGG
AATATACTAAGGGTACTGTTGACATTGCGAAGAGCGACAAAGATTTTGTTATCGGCTTTATTGCTC
AAAGAGACATGGGTGGAAGAGATGAAGGTTACGATTGGTTGATTGTGACACCCGGTGTGGGTTT
AGATGACAAGGAGACGCATTGGGTCAACAACAGTATAGAACCGTGGGATGATGTGGTCTCTACA
GGATCTGACATTATTATTGTTGGAAGAGGACTATTTGCAAAGGGAAGGGATGTGCTAAGGTAGA
GGGTGAACGTTACAGAAAAGCAGGCTGGGAAGCATATTTGAGAAGATGCGGCCAGCAAAACTA
AAAACTGTATTATAAGTAAATGTACGTATACTAAACTCACAAATTAGAGCTCTAATTTAATTTAT
CAGTTATTACCCTG

TGAGAGTGCACCACGCTTTCAATTCATCATTTTTTATTCTTTTTGATTTCGGTTTCGAAATTTTTTTG
ATTCGGTAATTCCGAACAGAAGGAAGAACGAAGGAAGGAGCATGACTTAGATTGGTATATATAC
ATATGTAGTGTTGAAGAAACATGAAATTGCCCAGTATTCTTAACCCAACTGCACAGAACAAAAA
CCTGCAGGAAACGAAGATAAATCATGTCGAGTTACATATAAGGAACGTGCTGCTACTCATCCTA
GTCCTGTTGCCGCTAAGCTATTTAATATCATGCACGAAAAGCAACAAATCCCAGGTAAGCCTATT
GGATGTTCGTACCACCAAGGCACTACTGGAGTTAAGTTGAAGCATTAGGTCCCAAAATTTGTTTA
CTAAAAACACATGTGGATATCTTGACTGATTTTTCCATGGAGGGGCACAGTTGCCGCTAAAGGCA
TTATCCGCCAAGTACAATTTTACTCGAAGACAGAAAATTTGCTGACATTGGTAATACAGTCAAAT
TGGCAGTACTTCTGCGGGTGTATACAGAATAGCAGAATGGGCAGACATTACGAACACACGGTGT
GGTGGGCCCAGGCATTGTTAGCGGTTTGAAGCAGGCGGCAGAAGAAGTAAATCGTAGCGAACCC
AGAGGCCTTTGATGTTAGTGCAGAATTGTCATGCAAGCGGCCCCTCGTATCTGGAGAATACTAAG
GGTACTGTTGACATTGCATTGTTTAATAAAGATTTTGTTATCGGCTTCATTGCTCAAAGACATTAC

GGGTGAAAAGAGATGAAGGTTACGATTGGTTGATTATGACACCCCGTGTGCTTTAGAATAGCCGC
GAGACGCATTGGGTCAAATTGCATAGAACCGTGGATGATGCGGTCTCTGCCATGATCTTGACATT
ATTGCTGGAAGAGGACCATTTGCAAAGGAAGGATGTGCTAAGGTAGAGGGTGAACGTTACAGA
AAAAGCAGGCTGGAAGCTATTATTTGAGAAGATGCGGCCAGCAAACCAAAAAACTGTATTATAA
GTAAATGCATGTATACTAAACTCACAAATTAATAGAGCTTCAATTTAATTATATATCAGTTATTAC
CCTA
chr6
TATTATATTAAATAAAATAATACACTGATTAACAATAAAGTTCATAAATAATTAAAATAATGATA
TTAATTACTTGTATTAATTACAAGCATATTTATACTCATCATCTTGGGCATTGAGAGGGAATCAAT
CAACTTCACAGCATGGGCAGATGTTCTATATGAAAATCAGACAAACAAAGCTAAGAGCAACGTA
CGC

Yellow highlight indicates the flanked the *ScURA3* fragment

Figure S8. Clone 5 Oxford Nanopore sequencing read
MiSeq (mi), Nanopore (nox)

Clone 15 PS10 intergenic-intergenic

AAAAAAAATTATTATGGAACAATTTGTGTG tgagagtgcaccaATTACGCTTTTCAATTC chr1 1098288 mi,nox

CAATTTAATTATATCAGTTATTACCCtg CATACGCGAAACTCAGGTGCTGCA chr5 1165171 mi,nox

CAATTTAATTATATCAGTTATTACCCtg
CATACGCGAAACTCAGGTGCTGCAATCTATATTTTTTTTTTTTTCCCTCTTACCATTCTGAAAAGACTTAAATAA

CAATTTAATTATATCAGTTATTACCCtg
CATACGCGAAACTCAGGTGCTGCAATCTATATTTTTTTTTTTTTTCCCT

chr5 1172530 single site Present in mi assembly

Nanopore monomer read 566 rc Both junctions are on same read.

Nanopore 086,454b,541b,566,629

>read566 reverse complement out2
TAGAACTAAATAAAATGTCTATAATGAGATTACACAAGGTATTAAACACAACCTTACCATTAAATATATG
AGTTAGATCTCCCAAATCATCGCGCACTTTTTCTTTTAATATCTTTTCATTTTTCCATACTAACTCTACTGCT
AAATGTAGTGAAGCAACAAAAAAAAATTATTATGGAACAATT **chr1**

TGTGTGATTACGCTTTTCAATTCATCATTTTTTTATTCTTTTTTTGATTTCGGTTTCCTTGAAATTTTTGATTC
GGTAATCTCCGAACAGAAGGAAGAACGAAAGCTGAAGCTGAGCACAGACTTAGATTGGTATATATACG
CATATGTAGTGTTGAAGAAACATGAAATTGCCCAGGCATTCTAACCCAAACAGAACAAAAACCTGCAGG
AAACGCAAAGAGTTAAATCATGTCGAAAGCTACATATAAGGAACGTGCTGCTACTCATCCTAGTCCTGTT
GCTGCCAAGCTGGGTTTAATATCATGCACGAAAAGCAAACAAACTTGTGTGCTTCATTGGATGTCGTACT
CACCAAGGAATTACTGAGTTAGTTGAAGCATTAGGTCCCAAAATTTGCTTTGCCAAACACATGTGGATAT
CTTGACTGATTTTTCCATGGAGGGCACAGTTAAGCTGCTAAAGGCATTATCCGTCATACAATTTTTTACTC
TTCGAAGACAGAAAAAATTTGCTGACATTGGTAATACAGTCAAATTGCAGTACCTGCGGGTGTATACAGA
ATAGCAGAATGGGCAGACATTACGAATGCACACGGTGTGGTGGGCCCAGGTATTGTTAGCGGTTTGAAG
CAGGCGGCAGAAGAAGTAACAAAGGAACCTAGAGGCCTTTTGATGTTAGCAGAATTGCCGCGCAAGGG
CTCCCCATCTACTGAGAATATACTAAGGGTACTGTTGATTGCGAAGAGCGACAAAGATTTGTTATCGGCC
ATTGCTCAAAGCATATATAAAGTGGGAAGAGATGAAGGTTACGATTGGTTGATTATGCACACCCGGTGTG
GCTTAGATGACAAGGGAGACGCATTGGGTCAACAGTATAGAACCGTGGATGATGTGGTCTCTACAGGAT
TCGACATTATTATTGTTGGAAGAGGACGGTTTGTAAAGGAAGGGATGCTAAGGTAGAGGGTGAACGTTA
CAGAAAAGCAGGCTGGGAAGCATATTTGAGAAGATGCGGCCAGCAAAACTAAAAACTGTATTATAAGT
AAATGCATGCATACTAAACTCACAAATTAGAGCTTCAATTTAATTATATCAGTTATTACCC
chr 5
CATACGCGAAACTCACAGGTGCTGCAATCTATATTTTTTTTTTTCCCTCTTACCATTCTTCTGAAAAGACTT
AACAATCATATAGGATAAGTACTGCAGCGGCTGTTCCTAAACAAATATGCAAGCCCACATAAGGCACGT
AGCACAATATTAATAGACCATATCTCTACTTAGTGAAAGTCATGAGCGGCTAAGAACG

Yellow highlight indicates the flanked the *ScURA3* fragment

Figure S9. Clone 15 Oxford Nanopore sequencing read

MiSeq (mi), Nanopore (nox)

Clone 23 PS8 SG4EUKG586913-intergenic

TATACACGAGATATTAGTCGAGTGGGA GTCCCATGCACCACGCTTTTCAA chr4 101355 mi
TTATATCAGTTATTACCCTG GTATCTTCGCTTTGTCTCTTAGCTTCC chr6 7292 mi

Nanopore monomer out2 read718, 421.067, 147, 164 (both ends),191,194(both ends),253,417(both ends),421(both ends),718(both ends), In chr6 partial on nox

> read164

TAGCCTTGTTGGCAAGATGGTATTATATACACACATGGCTAATAGTGTGCATCCGGTGGAGATTA
GGTACAGTGGAAAGTCGGATAACTCCGAAAACCGGAGACCCATGGAACGTCATCTCGCCATCGG
TGTCGCTGAACAGCTGCAATGAGTATATACACGAGATATTAGTCGAGTGGGA **chr4**

ATTCTATGCACCACGCTTTTCAATTCATCATTTTTTTATTCTTTTTTGATTTCGGTTTCCTTGAAAT
TTTTTGATTCGGTAATCTCCGAACAGAAGGAAGAACTTTGAGAGAATACAGACTTAGATTGAGTA
TATACGCATATGTAGTGGTGAAAACATGAAATTGCCCAGTATTAATAGCGCTACTGGGAACAAA
ACCTGCAGGAAACGAAGATAAATCATGTCAGCTACATATAAGGAACGTGCTGCTGTCATCCTAG
TCCTGTTGCTGCCAAGCTATTTAATATCATGCACGAAAAGCAAACAAACTTGTGTGCTTCATTGG
ATGTTCGTACCACCAAGGGAATTACTGGAGTTAGTTGAAGCATTAGGTCCCAAATTTGTTTACTA
AAACACATGTGGATGTCATGACTGATTTTTTCCATGGAGGGCACAGTTAAGCCGCTGAGGCATTA
TCCGCCAAGTAAATTTTTTACTCTTCGAAGACAGAAAATTTGCTGACATTGGTAATACAGTCAAA
TTGCAGTACTCTTGCGGGTGTACCTAGAATAGCAGAATGGGCAGACGTACGAATGCACACGGTG
TGGTGGGCCCAGGTATTGTTAGCGGTTTGAAGCAGGCGGCAGAAGTAACAAAGGGAACCTAGAG
CCTTTTGATGTTAGCAGAATTGTCATGCAAGGGGCTCTATACTGGAGAATATACTAAGGGTACTG
TTGACATTGCGAAGAGCGACAAAGATTTTGTTATCGGCTTTATTGCTCAAAAGAGACATGGGTGG
AAGAGATGAAGGTTACGATTGGTTGATTATGACACCCGGTGTGGGTTTTAGATGACAAGGGCGC
GTTAGGTGCGGCTTAGTAGAACCGTGGATGATGTGGTCTCACCACCCAGGATCTGACATTATTAT
TATTTAGGAAGGACTGTTGCGAAAGGGATGCTAAAGGTAGAGGTGAACGCGTTACAGAAAAGC
AGGCTGGGAGCATATTTGAGAAGATGCGGCCAGCAAAACTAAAACTGTATTATAAGTAAATGCA
TGTATACTAAACTCACAAATTAGAGCTTCAATTTAATTATATCAGTTATTACCCTG

GTATCTTCGCTTTGTCTCTTAGCTTCCATGTAAAGTGGTTACAAGTAACTAGAAACCAAAACTCAA
ACAAATAAAACCACAAACTTCAGAAACAGAACTTTCAAGTTGAAAAAGTTCTCCTCTCTTTTCTC
CTTCTATACTTTTAATTGGAGAAAGATATTTGGTATTTCAATTTGATTGACCTCTGGTTTTGCTTGA
TA **chr6**

Yellow highlight indicates the flanked the *ScURA3* fragment

Red indicates sequence from the read is different from *ScURA3* fragment

Figure S10 Clone 23 Oxford Nanopore sequencing read

MiSeq (mi), Nanopore (nox)

Primer name	Sequence 5'→3'
P1	GTATAGACGCATGAAGTCCTTCATTTGCTTTTGTTCCACA
P2	gagttctccgagaacaagcagaggttcgagTGTACTCGGA
P3	ctcgaacctctgcttgttctcggagaactcCCTCCTTTGATTAGTTTATGGGGCTAGCTG
P4	TAGGTGCCTGTCACGGCTCTTTTTTACTGTACCTGTGAC
P5	TTGGAAGTTAGCTTAGCAGAGGTAAAGTCG
P6	GGAAAGACCATTCTGCTTTACTTTTAGAGC
P7	atatcctaaattgcatgagggtgagATGTCGAAAGCTACATATAAGGAACGTGCTGCTAC
P8	tgccatgtaaaaacatacggatagTTAGTTTTGCTGGCCGCATCTTCTCAAATATGCTTC
P9	CAGCAAAACTAActatccgtatgtttttacatggcaattcctaattttgcaatcatgcac
P10	aggcgaggaagaggaaggtggaaatgttgaaaagtttg
P11	ACCTTCGAACCAACCatatcctaaattgcatgagggtgag
P12	gatgtactgcattcaatgacggttttggctcttccttcgtatttaacgggtttcga
P13	CAATGGTTGGGCATTGCAATTCCTACGTTCCTAATTAGTTTCATAGGATATGTTGCCCAC
P14	tatacgttctcaaaactcagagatggtgatgaaggcgaggaagaggaag
P15	GCATGAGGGTGAGcatggcaattcctaattttgcaatcatgcacaaatgatcgctag
P16	gcaaaattaggaattgccatgCTCACCCTCATGCAATTTAGGATATGGTTGGTTCGAAG
P17	CACAAACTTCATAAGCGTTCCCAAGGTTCGACC
PS1a	GCTAAGCTGTTGCAATGACCCAC
PS1b	CCCCGGTTCTTATATTTTCCCTATTACTACA
PS2a	TCTTCAATACCAAATTCGTCGTCTAGAG
PS2b	TTGTTTGGCTCCTTTGGTGAAGG
PS3a	CCTATTGAGAAAAAAACCGAAACTATTT
PS3b	GCTTAGAGTGTGTTATTTAGTCAAGAATTT
PS4a	TGATGATGATGATAAGATAGTGAAGAAGG
PS4b	TCATTTTACTTTCCAAACAAGACTAGATTCT
PS5a	TCCAAAGTCAAACCTTCCAAGTC
PS5b	CCTTTTTGACCTGTATAAATTTGCATTAC
PS6a	GGAATTTGCCAAGTCCAGAGG
PS6b	GCTGCATATCCACTACTGCTGC
PS7a	AATCAGAATCTTGAAGAGGAAAAG
PS7b	GACTTCCCCCCCTCAT
PS8a	TATACACGAGATATTAGTCGAGTGG
PS8b	GGAAGCTAAGAGACAAAGCG
PS9a	AAAACCAAAAAAAACCATAAAAACC
PS9b	CTATGTACATCATCAAGTCGTT
PS10a	AAAAAAAATTATTATGGAACAATTTGTGTGAT
PS10b	TGCAGCACCTGAGTT
PS11a	TGTGGCTAAGGTAACGG
PS11b	ACCAAGTGACCTGGC
PS12a	TGAAAAAATGATCCTGAAAAGA
PS12b	GGTGTTGCATGTGAC

PS13a	GATATACCAGTACAAAGGTAGTGA
PS13b	CGCTTCGTCCGTTTC
PS14a	TACAGTGGAAAGTCGGATAACT
PS14b	ACTGAGCTGACTGCATG
PS15a	ACCACTGTTACGTCGG
PS15b	TTTGTATAAGAAGACCTTGCG
PS16a	TTTTTCGATTTAATTAATATATATTATATAACAAC
PS16b	CAAACTATCGTCTTGGTATCTTTA
PS17a	CATGCCGGCCGAGTAACCGAGAGAA
PS17b	CGCATAAGAATCGATCCGA
PS18a	TGTACTTCTACGAAGGGC
PS18b	GAAAAAAAAAATAAAACTTTTTTTTCTCAAAAGCT
PS19a	ACTTCTAAATCGGATAGATTCGA
PS19b	ACTCTGGCATTTAATTTTGAGTC
PS20a	GGCGTCGGGGTAACTA
PS20b	CGAAAAAGAAACGCCCTCAG
PS21a	CCCTGCCGTATATACCTG
PS21b	GATCTGGGATTTAAGAGGCG
PS22a	CAATAAAACACTAAAATAAAATAAAATAAATTGAGAAAAAATCGA
PS22b	TTTTTCCATTATTTTTTTATATTATATTAGATATTTCCATGC
PS23a	CGACATTTTTTTTCGATTTAATTAATATATATTATATAACAAC
PS23b	CAAACTATCGTCTTGGTATCTTTAG
PS24a	TGTGGCTAAGGTAACGG
PS24b	ACCAAGTGACCTGGC
PS25a	GCCGTAACCTCTTTTTTATTG
PS25b	CGGAAAAAAAACGAAAAAAAAAAAAAAAAATA
PS26a	TTCAAAGTGGAAAAAAAACACCA
PS26b	ACGATTTCACAGGATTATCTAACG
PS27a	CATTAGCACCAAACCCACG
PS27b	TTGAGACCTGGTGTTTTATTAGGA
PS28a	TCCAAAGTCAAACCTTCC
PS28b	TTTGACCTGTATAAATTTGCATTAC
PS29a	GGAATTTGCCAAGTCCAGA
PS29b	GCTGCATATCCACTACTGC
PS30a	ACCGTGCATTAACGGT
PS30b	GGAAGAATCCGAGACACT
PS31a	TCTCAATTTATTTTATTTTATTTTAGTGTTTTATTG
PS31b	GGAAATATCTAATATAATATAAAAAAATAATGGAAAAA
PS32a	GCATCATTAGTAGTTATTGCATGG
PS32b	ACTCATTCCACTAGAATCTTCTCA
PS36a	TTTTCAAAGAACCGTGCATTAAC
PS36b	TGGGCAAGTTTTCCCG
PS37a	TGTACTTCTACGAAGGGC
PS37b	GGAATTATAAACACTTACATACGCTGA
PS283top	TTGGATCATGGATCTGACGTATTC
PS283bottom	CTAGCTGTCATCGGACAAATAAACTAAAAG

PS4-6F	TACAGTGGAAAGTCGGATAACT
PS4-6R	AGAATGGTGTGATTTAGGTTTCG
PSchr4delF	CCTGCAAATTACGCCTTC
PSchr4delR	GCCATACCAAGTGCG
PSade1F	CCAAGGTATACGTTGGTATA
PSade1R	CTATTACTCGTTGGTTCACTAG
PSadenewF	AGGAACTATCGTTAAAACCAAGGTATACGTT
PSadenewR	GACTTGGATTTGTATAAGTATTCCACTTCCCA
PSchr8a	ACTTCTAAATCGGATAGATTCGA
PSchr8b	ACTCTGGCATTTAATTTTGAGTC
PScl5a	TTCGGAATATTGAATTAATCGTCTCC
PScl5b	AATTAATATTATTTTTATTTAATATAATATAATTATTTATGAACTTTATTGTTAATC
T33	CAGAAACGGGAAGAATCTG
T34	TTCAACACCGTCGACA
T35	CTAGGCTATCACACACACTA
ura3-F	TGAGAGTGCACCACGCTTTTCAATTC
ura3-R	CAGGGTAATAACTGATATAATTAAATTG
ura3+720	GGGAAGGGATGCTAAGGTAGAGGGT
ura3+720c	ACCCTCTACCTTAGCATCCCTTCCC
URA3-R1-1-DN	CGGCCAGCAAAACTAAAAAACTG
ura3+61c	GCTTGGCAGCAACAGGACTAGGATG
ura3_TestF	GTTAGCGGTTTGAAGCAGGC
ura3_TestR	GCCTGCTTCAAACCGCTAAC

Table S1. Primers used in PCR reactions. F: forward. R: reverse. DN: downstream. chr: chromosome. Del: deletion. Lower case letters such as a, c, and c are given as a way to keep tracking the number of primers for each testing locus.
